# TRPM7 Kinase and Mitochondrial Dysfunction in Diabetic Heart Failure With Preserved Ejection Fraction: A Molecular Entry Point to a Complex Disease

**DOI:** 10.1016/j.jacbts.2025.101350

**Published:** 2025-07-24

**Authors:** Norimichi Koitabashi

**Affiliations:** Department of Cardiovascular Medicine, Gunma University Graduate School of Medicine, Maebashi, Japan; Division of Cardiology, Gunma Prefectural Cardiovascular Center, Maebashi, Japan

**Keywords:** diabetes, HFpEF, magnesium, TRPM7, mitochondria

**Figure:**
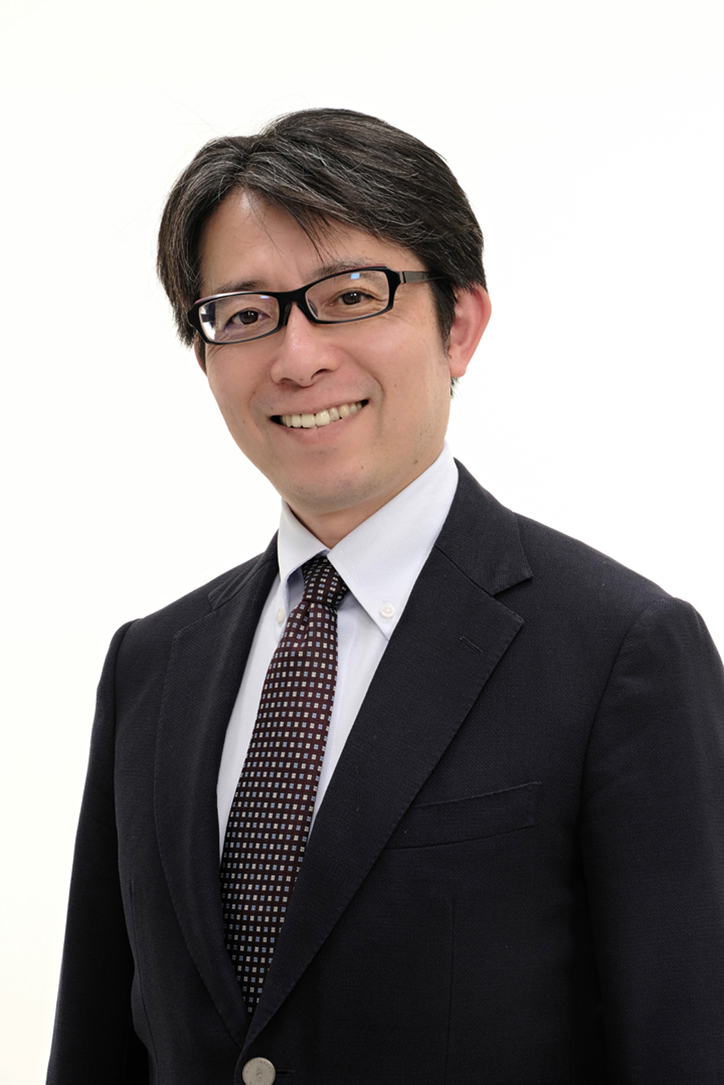

Heart failure with preserved ejection fraction (HFpEF), particularly in individuals with diabetes mellitus, has become a significant public health concern. Although recent therapeutic advances have improved outcomes for patients with heart failure with reduced ejection fraction, effective treatments for HFpEF remain limited.^1^ In particular, diabetic HFpEF is characterized by systemic inflammation, endothelial dysfunction, and metabolic derangements, including impaired mitochondrial energetics.^2^^,^^3^ This has sparked growing interest in elucidating the cellular mechanisms that link metabolic stress with cardiac dysfunction. In this context, the new study by Liu et al,^4^ published in this issue of *JACC: Basic to Translational Science*, provides critical insight into the role of transient receptor potential melastatin 7 (TRPM7) kinase in mitochondrial dysfunction and diastolic impairment in a preclinical model of diabetic HFpEF.

Liu et al^4^ demonstrate that TRPM7 kinase, not its ion channel function, mediates mitochondrial oxidative stress and subsequent cardiac dysfunction in diabetic conditions. Using TRPM7 kinase-dead knock-in mice fed a high-fat or low-magnesium diet, the authors observed attenuated diastolic dysfunction and reduced mitochondrial production of reactive oxygen species compared with wild-type controls. In a prior study, they showed that high-fat diet–induced diabetic mice develop hypomagnesemia, and that dietary magnesium supplementation improves both mitochondrial function and diastolic performance.^5^ In the current work, the use of a low-magnesium diet further exacerbated magnesium deficiency, allowing for a more precise delineation of TRPM7 kinase’s pathologic role. These findings strengthen the mechanistic link between magnesium deficiency, mitochondrial dysfunction, and diastolic impairment in diabetic hearts. Mechanistically, TRPM7 kinase activation was found to upregulate a Src family tyrosine kinase, leading to mitochondrial complex II inhibition and reactive oxygen species accumulation. Notably, supplementation with magnesium sulfate ameliorated these effects, highlighting the central role of magnesium homeostasis in TRPM7-mediated cardiac injury.^4^

The role of transient receptor potential (TRP) channels in cardiovascular pathophysiology is increasingly recognized. TRPM7 is unique in possessing both ion channel and serine/threonine kinase activities, allowing it to sense and integrate magnesium levels and cellular stress signals. We and other groups have shown that another TRP channel, TRPC6, contributes to pathologic cardiac hypertrophy via nuclear factor of activated T-cells-dependent signaling pathways, highlighting the TRP family members as versatile regulators of myocardial remodeling.^6^ However, TRPM7 appears to operate through a distinct, mitochondrial-centric mechanism that is particularly relevant in metabolic cardiomyopathy. This mechanistic divergence underscores the broader significance of TRP channels in cardiovascular disease, where different subtypes may drive distinct aspects of the pathologic remodeling process.

A key strength of the study lies in its use of a kinase-dead TRPM7 model, which allows for clear separation of kinase function from channel activity.^4^ The authors provide compelling in vivo evidence supported by isolated mitochondrial and molecular analyses. Furthermore, by linking magnesium handling with oxidative phosphorylation, the study extends our understanding of how metabolic stressors exacerbate cardiac dysfunction.^4^ This is particularly relevant given the growing clinical recognition of hypomagnesemia as a modifiable risk factor in patients with diabetes mellitus and heart failure.^7^^,^^8^

The translational relevance of this study is underscored by the growing interest in targeting mitochondrial dysfunction in HFpEF. Current pharmacologic strategies (eg, glucagon-like peptide-1 receptor agonists, sodium-glucose cotransporter 2 inhibitors) have demonstrated benefits in diabetic patients, some of which may be mediated by improvements in mitochondrial health and magnesium balance.^7^^,^^9^ Whether these therapies modulate TRPM7 kinase activity remains an open question, but the present findings suggest potential synergy. In addition, targeting TRPM7 directly or its downstream effector a Src family tyrosine kinase could represent a novel therapeutic avenue. The development of specific TRPM7 kinase inhibitors, or approaches to restore magnesium homeostasis in cardiac tissues, may offer new treatment strategies for a condition that has largely eluded effective intervention.

Although Liu et al^4^ demonstrate a mechanistic role for TRPM7 kinase-mediated mitochondrial dysfunction in a murine model of diabetic HFpEF, the translational relevance of magnesium signaling in human heart failure remains to be clarified. Clinical trials have shown that SGLT2 inhibitors not only improve outcomes in HFpEF patients, but they also increase serum magnesium levels.^7^ However, the extent to which magnesium modulation contributes to the clinical benefits of SGLT2 inhibitors is not fully understood. Moreover, in a large clinical cohort of patients with myocardial infarction and left ventricular dysfunction from the EPHESUS (Eplerenone Post-Acute Myocardial Infarction Heart Failure Efficacy and Survival Study) trial, Martens et al^10^ found that neither hypomagnesemia nor hypermagnesemia was independently associated with adverse cardiovascular outcomes. This suggests that serum magnesium levels may not be reliable biomarkers for intracellular magnesium status or for guiding therapy in heart failure. These conflicting findings highlight the need for further translational studies examining magnesium homeostasis at the tissue level, specifically focusing on intracellular magnesium dynamics and TRPM7 activity in human HFpEF populations.

Despite its strengths, the study has limitations. The use of only male mice limits generalizability, especially given known sex differences in HFpEF prevalence and pathophysiology. The sample size for hemodynamic assessment was relatively modest, and although mitochondrial outcomes were well characterized, complementary measures (eg, titin isoform shifts, extracellular matrix remodeling) were not fully explored. Finally, whereas murine models provide valuable mechanistic insight, confirmation in human cardiac samples or patient-derived cardiomyocytes would strengthen the translational potential of these findings.

In conclusion, Liu et al^4^ identify TRPM7 kinase as a novel upstream mediator of mitochondrial dysfunction and diastolic impairment in a diabetic HFpEF model. Their work broadens our understanding of magnesium-dependent signaling in metabolic heart failure and establishes a potential therapeutic target within the TRP channel family. As precision medicine approaches gain momentum in HFpEF, targeting specific molecular drivers (eg, TRPM7 kinase) may offer a path toward more effective and individualized therapies.

## Funding Support and Author Disclosures

This study was supported by the Japan Society for the Promotion of Science (25K11293). The author has reported that they have no relationships relevant to the contents of this paper to disclose.
